# Differential expression of innate and adaptive immune genes in the survivors of three gibel carp gynogenetic clones after herpesvirus challenge

**DOI:** 10.1186/s12864-019-5777-z

**Published:** 2019-05-28

**Authors:** Wei-Jia Lu, Fan-Xiang Gao, Yang Wang, Qi-Ya Zhang, Zhi Li, Xiao-Juan Zhang, Li Zhou, Jian-Fang Gui

**Affiliations:** 1 State Key Laboratory of Freshwater Ecology and Biotechnology, Institute of Hydrobiology, The Innovation Academy of Seed Design, Chinese Academy of Sciences, Wuhan, 430072 Hubei China; 2 University of Chinese Academy of Sciences, Beijing, 100049 China; 3 Institute of Marine Biology, College of Oceanography, Hohai University, Nanjing, 210098 China

**Keywords:** Gibel carp, Herpesvirus, Transcriptome, Survivors, IFN, Immunoglobulin, Myosin

## Abstract

**Background:**

Accompanied with rapid growth and high density aquaculture, gibel carp has been seriously threatened by *Carassius auratus* herpesvirus (*Ca*HV) since 2012. In previous study, distinct *Ca*HV resistances and immune responses were revealed in the diseased individuals of three gibel carp gynogenetic clones (A^+^, F and H). However, little is known about the gene expression changes in the survivors after *Ca*HV challenge, particularly their differences of innate and adaptive immune system between susceptible clone and resistant clone.

**Results:**

We firstly confirmed the *Ca*HV carrier state in the survivors of three gibel carp clones after *Ca*HV challenge by evaluating the abundances of five *Ca*HV genes. The assay of viral loads indicated the resistant clone H possessed not only stronger resistance but also higher tolerance to *Ca*HV. Then, 2818, 4047 and 3323 differentially expressed unigenes (DEUs) were screened from the head-kidney transcriptome profiles of survivors compared with controls from clone A^+^, F and H. GO and KEGG analysis suggested that a persistent immune response might sustain in resistant clone H and F, while susceptible clone A^+^ had a long-term impact on the circulatory system which was consistent with the major symptoms of bleeding caused by *Ca*HV. Among the top 30 enriched pathways of specifically up-regulated DEUs in respective clones, 26, 7 and 15 pathways in clone H, F and A^+^ were associated with infections, diseases, or immune-related pathways respectively. In addition, 20 pathways in clone F belonged to “metabolism” or “biogenesis”, and 7 pathways involved in “circulatory system” were enriched in clone A^+^. Significantly, we revealed the differential expression changes of IFN system genes and immunoglobulin (Ig) genes among the survivors of three clones. Finally, myosins and Igs were identified as co-expression modules which were positively or negatively correlated to *Ca*HV viral loads respectively.

**Conclusions:**

Our results revealed the common and distinct gene expression changes in immune and circulatory system in the survivors of three gibel carp gynogenetic clones with different *Ca*HV resistances. The current study represents a paradigm of differential innate and adaptive immune reactions in teleost, and will be beneficial to the disease-resistance breeding of gibel carp.

**Electronic supplementary material:**

The online version of this article (10.1186/s12864-019-5777-z) contains supplementary material, which is available to authorized users.

## Background

Gibel carp (*C. gibelio*), as a natural polyploid fish [1, 2], widely distributes across the Eurasian continent [3–9] and is able to reproduce by unisexual gynogenesis or sexual reproduction [10–16]. Recently, we discriminated numerous various gynogenetic clones of gibel carp from 35 locations through mainland China by transferrin allele and mtDNA haplotype polymorphism [5, 6]. Along with the large-scale application of several improved varieties, all-female allogynogenetic gibel carp activated by heterologous sperm has become one of the most important farmed fish in China [17]. The annual production capacity has increased to more than 2.82 million tons in 2017 [18]. However, the highly intensive farming has resulted in serious disease problems. Especially, the infections of herpesvirus *Ca*HV in crucian carp (*Carassius auratus*), a newly emerged epizootic disease with acute gill hemorrhages and high mortality, has outbroken in main culture areas of Jiangsu province and caused huge economic losses since 2012 [19].

Herpesviruses are widespread among vertebrates, some of which caused devastating diseases in aquaculture [20–25]. One of effective ways to reduce the threat is to breed resistant strains or crossbreeds to viral infection [26]. In our previous study, we revealed the distinct resistances to herpesvirus *Ca*HV among three gynogenetic clones of gibel carp through herpesvirus *Ca*HV challenge experiments. Allogynogenetic gibel carp “CAS III” (clone A^+^) [27], the most popularly cultured variety in China, was a highly susceptible clone. The novel variety (clone F) [28], nominated as the allogynogenetic gibel carp “CAS V”, showed moderately resistant to *Ca*HV. We also identified a wild clone H as a strongly resistant clone [29]. Our results confirmed that the ability of fish to resist infection was strongly influenced by genetic factors [30–34].

The first step towards disease-resistance breeding is the search for associations between disease resistance and molecular markers. Teleost possesses complicated innate immune network and primitive adaptive immune system [35–37]. When the pathogen invaded, the innate immune system provides the immediate sensing and elimination of pathogens in a nonspecific and nonmemory manner, while the adaptive immune system offers delayed and highly specialized responses with immunologic memory features [38, 39]. Through transcriptome analysis among control and diseased individuals from clone A^+^, F and H, we revealed that resistant clone H of gibel carp activated stronger innate interferon (IFN)-mediated antiviral response, while susceptible clone A^+^ lost the battle with *Ca*HV and evidently induced apoptosis or death [29]. Eight IFN system genes, exhibiting sharply up-regulated and higher expression in resistant clone H than those in susceptible clone A^+^ and F at 1 or 3 days post infection (dpi), were identified as candidate resistant-related genes [40]. However, little is known about the gene expression changes in the survivors after *Ca*HV challenge, particularly their differences of innate and adaptive immune system responses between susceptible clone and resistant clone. In this study, we first confirmed the carrier state in survivors of three gibel carp clones after *Ca*HV challenge. Then, we revealed the common and distinct gene expression changes in immune and circulatory system in the survivors of gibel carp three clones by comprehensive transcriptomic analysis and qPCR. Finally, we performed weighted gene correlation network analysis (WGCNA) to identify the co-expression modules correlated to resistant traits.

## Results

### Confirmation of herpesvirus *Ca*HV carrier state in gibel carp survivors after *Ca*HV challenge

**Fig. 1:**
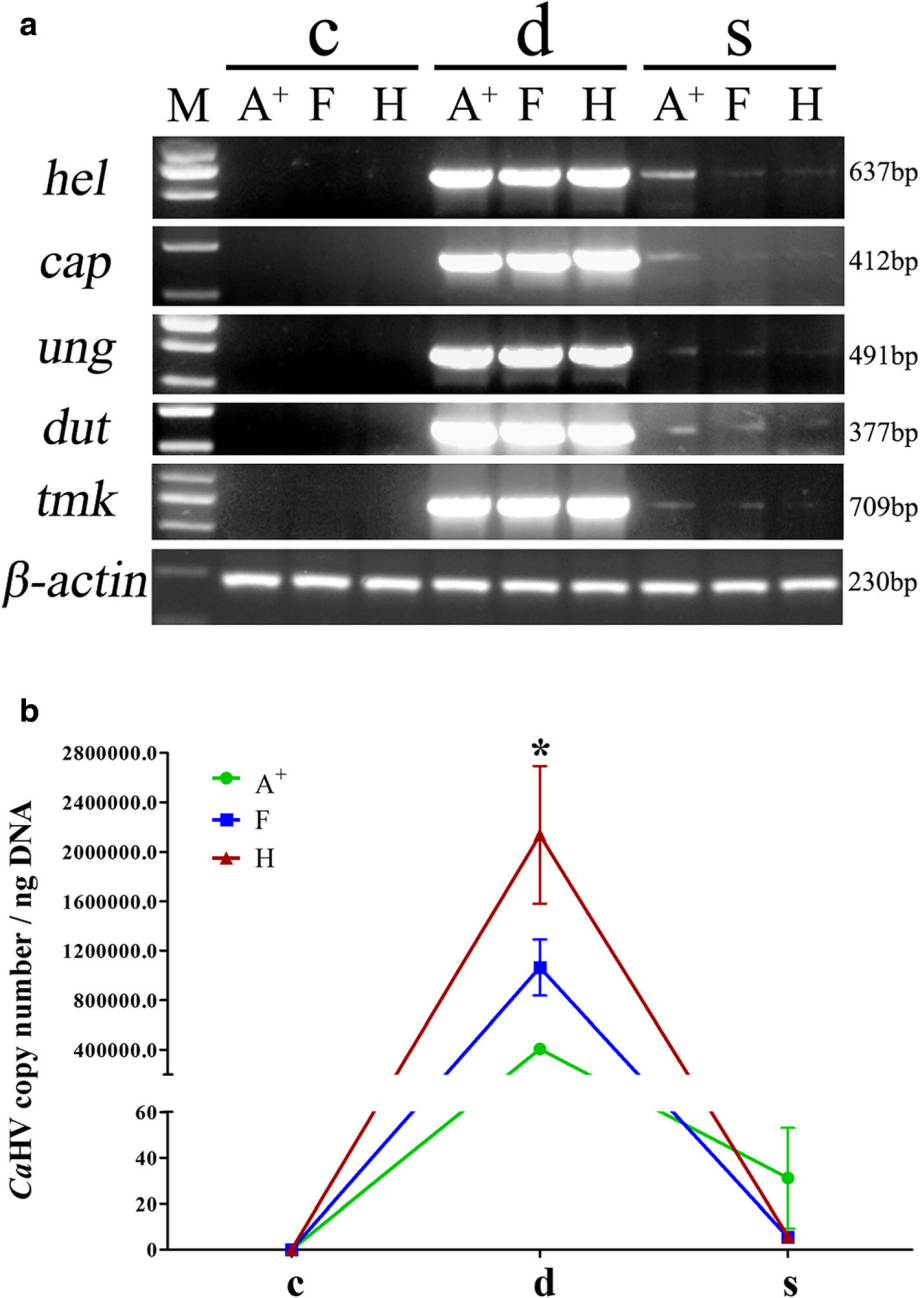
*Ca*HV detection and quantification in the control individuals (c), diseased individuals with sub-clinical symptoms (d) or survivors (s) from three gynogenetic gibel carp clones (A^+^, F and H). **a** Electrophoretogram of the PCR amplified products of five *Ca*HV genes (*hel*, *cap*, *ung*, *dut* and *tmk*). β-actin was selected as reference gene. Gene symbols and the sizes of amplified products are indicated by the left and right side of the figure, respectively. **b** *Ca*HV viral loads. Data are shown as means ± standard deviation (SD) (*n* = 3) representing *Ca*HV copy number per ng DNA. The asterisk indicates the significant difference between resistant clone and susceptible clone (*p* < 0.05)

The three gynogenetic gibel carp clones (A^+^, F and H) exhibited significantly different resistance to *Ca*HV [29]. The mortalities caused by *Ca*HV began at 3 dpi and subsided after 14 dpi, resulting in cumulative mortalities of 98.89, 86.67 and 51.11% in clone A+, F and H. The survivors appeared healthy at 28 dpi. We first tested the abundances of five *Ca*HV genes, including *helicase-primase helicase subunit* (*hel*), *capsid triplex subunit 2* (*cap*), *uracil-DNA glycosylase* (*ung*), *deoxyuridine triphosphatase* (*dut*), and *thymidylate kinase* (*tmk*) in the control individuals, diseased individuals and survivors to evaluate the *Ca*HV carrier state (Fig. 1a). After 40 amplification cycles, no DNA fragment was amplified from the control individuals (c), while strong or weak bands were amplified in the diseased individuals with sub-clinical symptoms (d) or survivors (s) respectively.

Then, the viral loads were evaluated by real-time PCR (Fig. 1b). The control individuals from three clones were confirmed to be *Ca*HV-free. A few of *Ca*HV particles were detected with loads of 10^1.27 ± 0.31^, 10^0.73 ± 0.07^, and 10^0.70 ± 0.16^ particles/ng DNA in survivors from clone A^+^, F and H respectively, implying that survivors had conquered the herpesvirus and eventually survived with *Ca*HV carrier state. Extremely abundant *Ca*HV particles were detected in the diseased individuals. Different from lower viral loads in resistant clone H than that in susceptible clone A^+^ during the early infected stage (1–5 dpi) [29, 40], higher viral loads were detected in the diseased individuals of clone H (10^6.29 ± 0.14^) than that in the diseased individuals of clone A^+^ (10^5.61 ± 0.02^) (Fig. 1b). These results further confirmed that the resistant clone H possessed not only stronger resistance but also higher tolerance to *Ca*HV.

### Stronger immune responses persisted in survivors of gibel carp resistant clone after *Ca*HV challenge

In our previous study, we analyzed the comparative transcriptome data between control and diseased individuals [29]. To better understand the interactive mechanisms between gibel carp and *Ca*HV, we further identified 2818, 4047 and 3323 differentially expressed unigenes (DEUs) (probability ≥0.8 and relative change ≥2) from the head-kidney transcriptome profiles of survivors (s) compared with control individuals (c) from clone A^+^, F and H (s-A^+^ vs c-A^+^, s-F vs c-F, and s-H vs c-H) respectively (Additional file 1: Table S1, Additional file 2 Table S2 and Additional file 3: Figure S1).

**Fig. 2:**
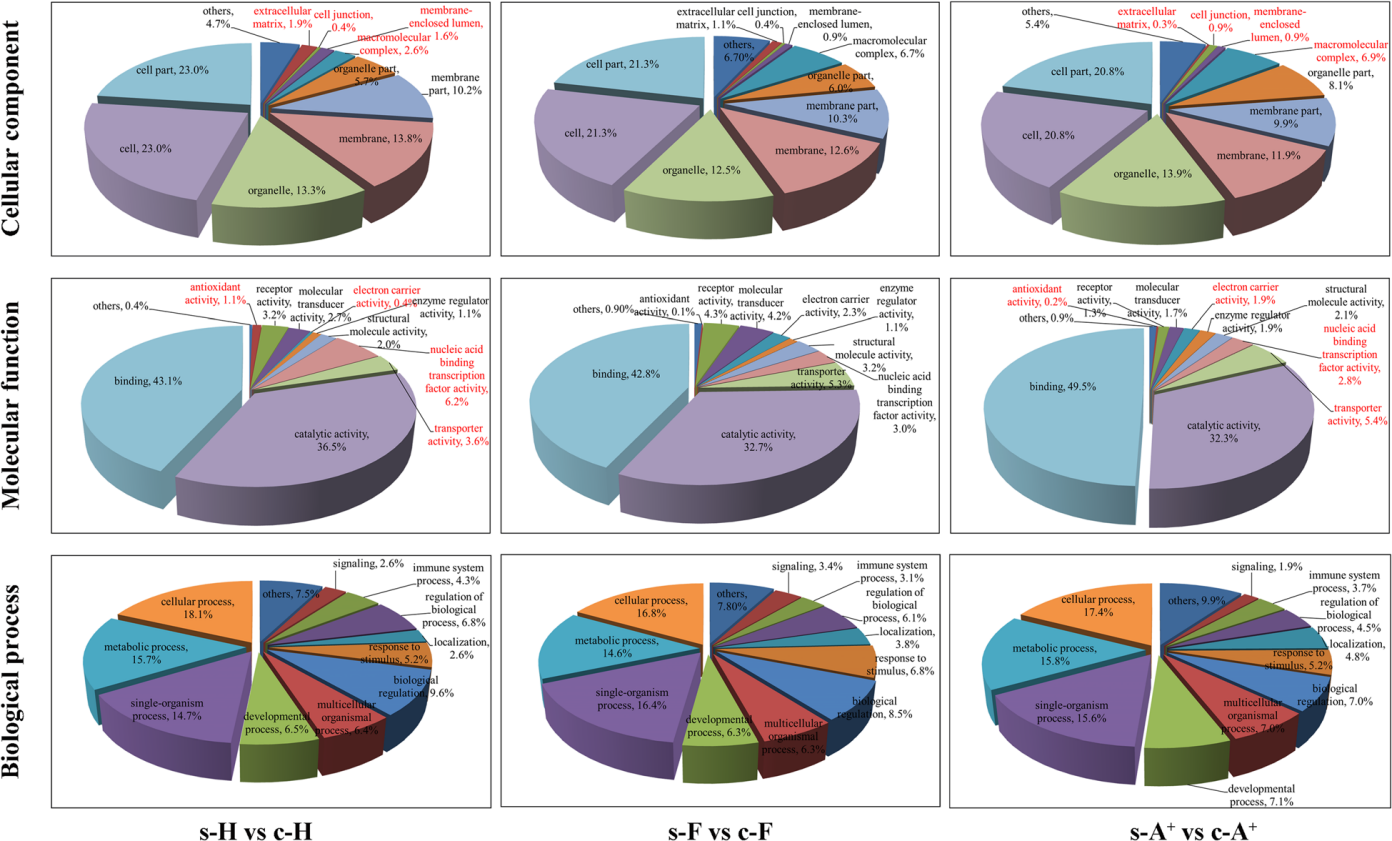
GO classification of DEUs from s-H vs c-H, s-F vs c-F and s-A^+^ vs c-A^+^, respectively. GO terms grouped into three main categories: biological process, cellular component and molecular function. The ID and percentage of each GO term are indicated in the pie chart

GO (Gene Ontology) analysis showed that the DEUs identified from s-A^+^ vs c-A^+^, s-F vs c-F and s-H vs c-H were categorized into 51, 51 and 47 terms with similar distribution patterns, respectively. Nevertheless, subtle differences of categories were found among three clones (Fig. 2). For cellular component, the percentages of DEUs in term “extracellular matrix” and “membrane-enclosed lumen” identified from resistant clone H were significant higher than those identified from susceptible clone A^+^, while remarkably less DEUs of clone H were distributed in the “cell junction” and “macromolecular complex” compared with clone A^+^. For molecular function, the rates of term “antioxidant activity”, “nucleic acid binding transcription factor activity”, “electron carrier activity”, and “transporter activity” exhibited noticeable differences between s-A^+^ vs c-A^+^ and s-H vs c-H.

**Fig. 3:**
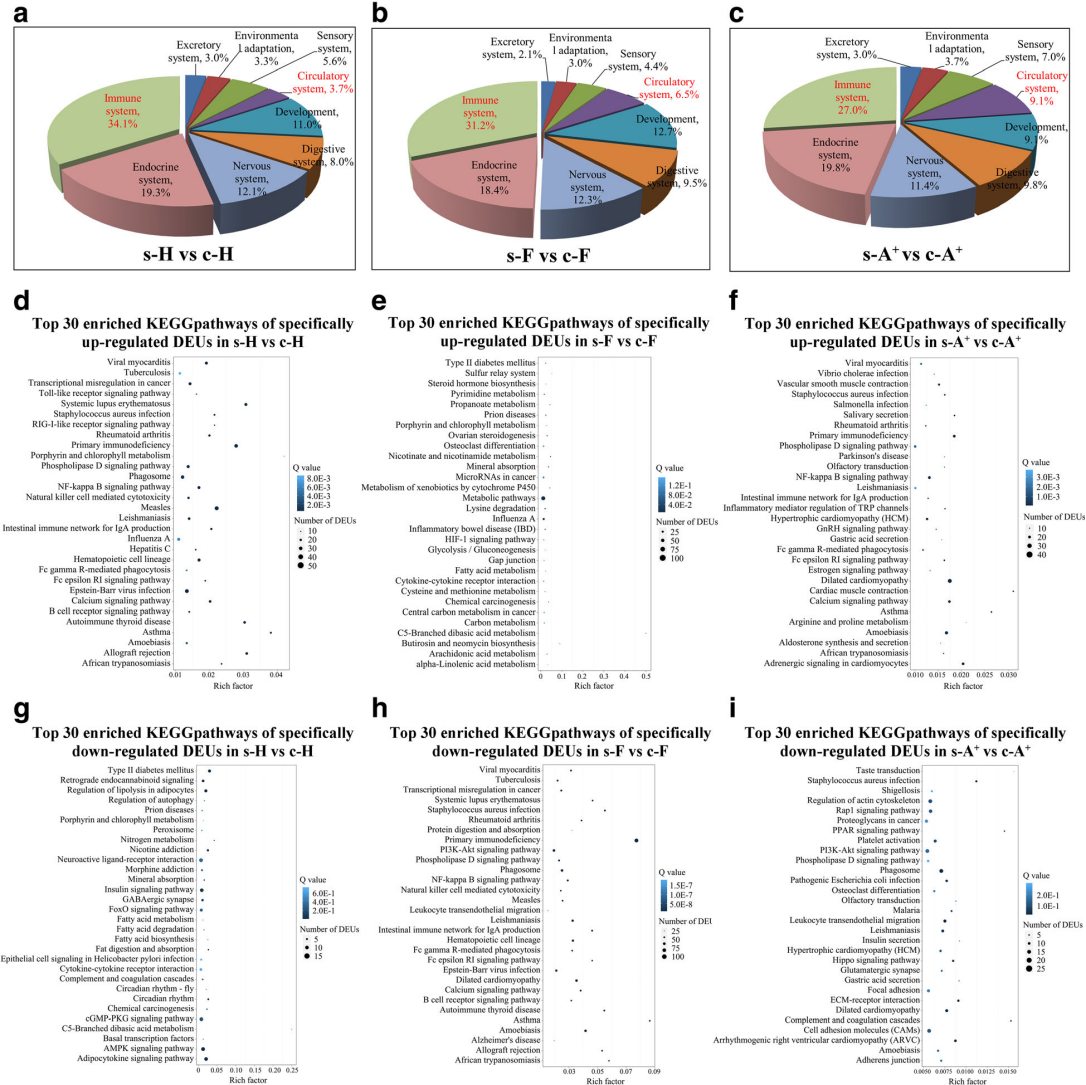
KEGG pathway enrichment analysis. **a-c** Functional classification of DEUs in KEGG term “organismal systems” from s-H vs c-H (**a**), s-F vs c-F (**b**) and s-A^+^ vs c-A^+^ (**c**). The ID and percentage of each KEGG term are indicated in the pie chart. **d-i** Top 30 enriched KEGG pathways of specifically up- or down-regulated DEUs from s-H vs c-H, s-F vs c-F and s-A^+^ vs c-A^+^, respectively. The x-axis indicates the rich factor of each pathway and y-axis shows pathway. The color and size of dot indicates Q value and the number of DEUs assigned to the corresponding pathway respectively

KEGG (Kyoto Encyclopedia of Genes and Genomes) pathway mapping revealed that 4, 9 and 8 pathways enriched in s-A^+^ vs c-A^+^, s-F vs c-F and s-H vs c-H were associated with “immune system”, “immune diseases”, “infectious diseases”, and “cardiovascular diseases” among the top 10 enriched pathways (Additional file 4: Figure S2; Additional file 5: Table S3). 570, 873, and 602 DEUs were categorized into “organismal systems”. The percentages of DEUs mapped into pathway “immune system” in resistant clone H (34.1%) and clone F (31.2%) was significantly higher than that in susceptible clone A^+^ (27.0%), while less ratios of DEUs mapped into pathway “circulatory system” in clone H (3.7%) and clone F (6.5%) were observed when compared to that in susceptible clone A^+^ (9.1%) (Fig. 3a-c). The results indicate that stronger immune responses persisted in the survivors of gibel carp resistant clone H after *Ca*HV challenge. In addition, the results also imply the obvious pathological changes in circulatory system occurred in susceptible clone A^+^, consistent with the main symptom of breeding in gibel carp after *Ca*HV challenge.

### Activation of circulatory and immune system in the survivors of all three gibel carp clones

**Fig. 4:**
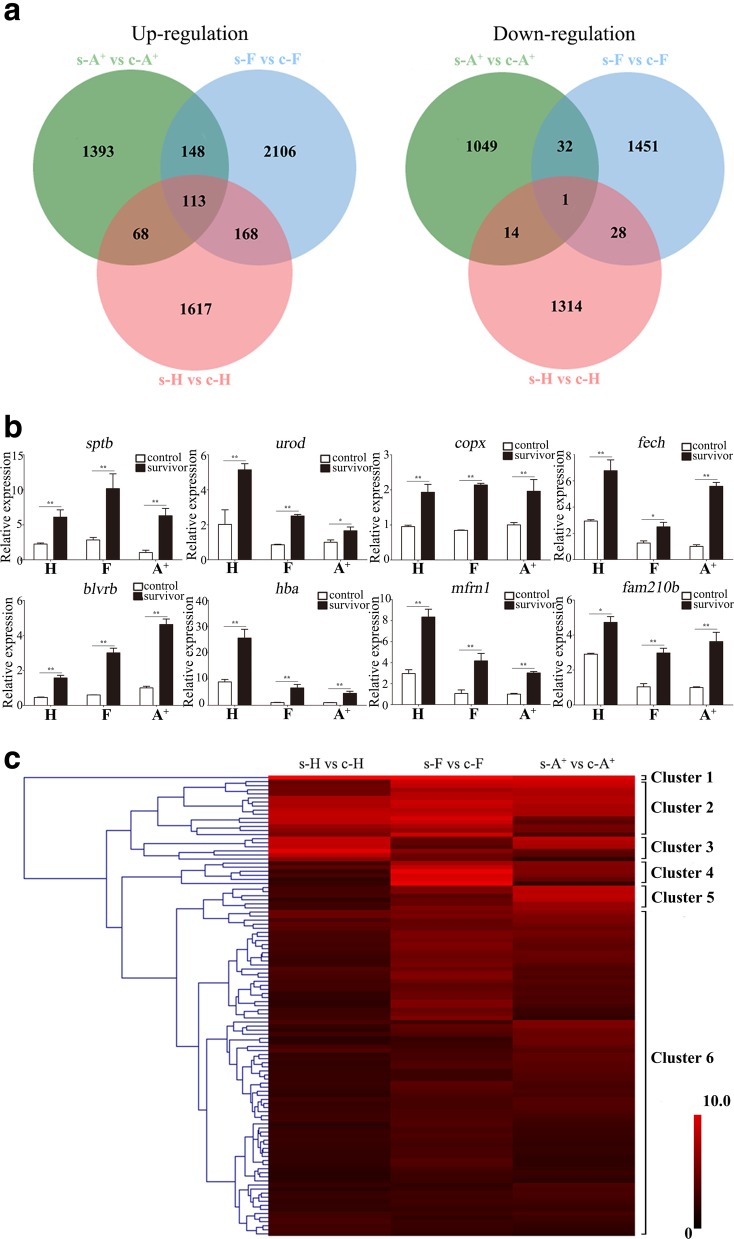
Commonly up-regulated DEUs from s-H vs c-H, s-F vs c-F and s-A^+^ vs c-A^+^. **a** Venn diagram of up- or down-regulated DEUs from s-A^+^ vs c-A^+^ (green circle), s-F vs c-F (blue circle) and s-H vs c-H (red circle). **b** The qPCR analyses of eight genes (*sptb*, *urod*, *cpox*, *fech*, *blvrb*, *hba*, *mfrn1* and *fam210b*) related to circulatory system in head kidney of gibel carp clone A^+^, F and H. *eef1a1l1* was used as the normalizer. Gene expression levels are relative to that of clone A^+^ control individuals. Each bar represents mean ± standard deviation (SD) (*n* = 3). Asterisks indicate the significant differences between control and survivor (*: *p* < 0.05 and **: *p* < 0.05). **c** Heatmap of commonly up-regulated DEUs among s-H vs c-H, s-F vs c-F and s-A^+^ vs c-A^+^. Hierarchical clustering is calculated by log_2_ fold change values and the main clusters were lined out on the right

Through integrating the shared DEUs among s-A^+^ vs c-A^+^, s-F vs c-F and s-H vs c-H, only 113 and 1 DEUs were up-regulated or down-regulated in all three clones obtained from the total of 5613 up-regulated DEUs and 3889 down-regulated DEUs (Fig. 4a; Additional file 6: Table S4). Among 113 commonly up-regulated DEUs, 49 DEUs are annotated as genes which are the membrane proteins of erythrocyte (*ank1* and *sptb*) or participate in heme biosynthesis and metabolism (*alas2*, *hmbs*, *urod*, *cpox*, *fech*, and *blvrb*), oxygen and ion transport (*hba*, *hbb*, *tfr*, *mfrn1*, *slc4a1*, and *cahz*), or proliferation and differentiation of blood cells (*hemge*, *klf1* and *fam210b*) (Table 1). We selected 8 genes, including *sptb*, *urod*, *cpox*, *fech*, *blvrb*, *hba*, *mfrn1*, and *fam210b*, and confirmed their commonly up-regulated expression in the survivors of all three gibel carp clones in comparison with control individuals by qPCR (Fig. 4b).

**Table 1:** Category of commonly up-regulated DEUs annotated in the circulatory system from s-H vs c-H, s-F vs c-F and s-A^+^ vs c-A^+^

Gene Name	Gene Abbrev-iation	Function annotation	Unigene ID
*Delta-aminolevulinate synthase*	*alas2*	Heme biosynthesis and metabolism	CL55.Contig1, CL55.Contig2, CL55.Contig3
*Ferrochelatase*	*fech*		CL8987.Contig5, CL8987.Contig6, Unigene35721
*Hydroxymethylbilane synthase b*	*hmbsb*		CL8862.Contig4, CL8862.Contig1, CL8862.Contig7, CL8862.Contig6, CL8862.Contig3, CL8862.Contig2
*Uroporphyrinogen decarboxylase*	*urod*		CL158.Contig2, CL158.Contig1, CL158.Contig3
*Coproporphyrinogen oxidase*	*cpox*		CL3455.Contig8
*Flavin reductase*	*blvrb*		CL8222.Contig4, CL8222.Contig2, CL8222.Contig5
*Hemoglobin alpha*	*hba*	Oxygen and ion transport	CL5869.Contig1, CL5869.Contig2, CL5869.Contig6
*Hemoglobin beta*	*hbb*		Unigene31300, Unigene86068, Unigene6944, Unigene22310,
*Transferrin receptor*	*tfr*		CL14300.Contig2, CL14300.Contig4, CL14300.Contig5, Unigene38629, CL14300.Contig1
*Mitoferrin-1*	*mfrn1*		CL45.Contig4, CL45.Contig1
*Band 3 anion transport protein*	*slc4a1*		CL6154.Contig1, CL6154.Contig4, CL6154.Contig3
*Carbonic anhydrase*	*cahz*		Unigene28427
*Hemogen*	*hemge*	Proliferation and differentiation of haemocytes	CL5210.Contig1, CL5210.Contig4, CL5210.Contig2, CL5210.Contig5, CL5210.Contig3
*Kruppel-like factor 1*	*klf1*		CL4920.Contig3, CL4920.Contig1
*Family with sequence similarity 210 member B*	*fam210b*		CL6436.Contig3,
*Ankyrin 1*	*ank1*	Erythrocyte membrane structure	Unigene12266
*Spectrin beta*	*sptb*		Unigene1305, CL16738.Contig5

Moreover, 21 DEUs were mapped to innate immunity (*gig2*, *ifi44, pla2g4b*, and *alox15*) or adaptive immunity (*klf13*, *stab1, spen*, *mycbp2*, *huwe1*, *nf1*, *birc6*, *klf17*, and *pikks*), and the last six genes were involved in cell proliferation and tissue repair (Table 2). These results indicate that *Ca*HV infection results in the activation of circulatory or immune system in the survivors of all three gibel carp clones. In addition, we also identified 10 highly up-regulated DEUs which were involved in vesicle transport or cycling, such as *vacuolar protein sorting-associated protein 13C* (*vps13c*), *sodium/bile acid cotransporter 4* (*slc10a4*) and *WD repeat and FYVE domain-containing protein 4* (*wdfy4*). Only one DEU (CL16220.Contig1_All) exhibited commonly down-regulated among three gibel carp clones and was annotated as *metallothionein* (*mt1*).

**Table 2:** Category of commonly up-regulated DEUs annotated in the immune system from s-H vs c-H, s-F vs c-F and s-A^+^ vs c-A^+^

Gene Name	Gene Abbrev-iation	Category	Unigene ID
*grass carp hemorrhagic virus-induced gene 2*	*gig2*	Innate immunity	CL5938.Contig2, CL5938.Contig1
*interferon-induced protein 44*	*ifi44*		Unigene19789
*cytosolic phospholipase A2 beta*	*pla2g4b*		CL1686.Contig3, CL1686.Contig18, CL1686.Contig14
*arachidonate 15-lipoxygenase*	*alox15*		CL1331.Contig11, CL1331.Contig10
*krueppel-like factor 13*	*klf13*	Adaptive immunity	Unigene33364, Unigene73086
*stabilin-1*	*Stab1*		CL3993.Contig1
*msx2-interacting protein*	*spen*		CL16106.Contig2
*E3 ubiquitin-protein ligase mycbp2*	*mycbp2*		Unigene17417, Unigene38807,
*E3 ubiquitin-protein ligase huwe1*	*huwe1*		CL4981.Contig5, CL4981.Contig1
*nuclear factor I*	*nf1*		CL14567.Contig1
*baculoviral IAP repeat-containing protein 6*	*birc6*		CL14135.Contig1
*kruppel-like factor 17*	*klf17*		CL10390.Contig2
*phosphatidylinositol 3-kinase-related kinase*	*pikks*		Unigene23119

Hierarchical clustering analysis showed that the commonly up-regulated DEUs were classified into six clusters (Fig. 4c). The cluster 1 contained only one DEU (CL11903.Contig1_All) which was not annotated in any of the public databases and displayed remarkably up-regulated (about 2^10^ folds) expression in the survivors of all three gibel carp clones compared to control individuals, while the cluster 6 included about two-thirds of DEUs which exhibited moderately and similarly up-regulated expression in the survivors of all three gibel carp clones. The cluster 3 and 5 represented the distinct responses to *Ca*HV between resistant clone H and susceptible clone A^+^. DEUs in cluster 3 showed higher increase levels in s-H vs c-H than those in s-A^+^ vs c- A^+^, and most of them involved in immune reaction, such as *mycbp*, *birc6*, *klf13* and *ifi44*. On the contrary, DEUs in cluster 5 displayed relatively higher upregulation in s-A^+^ vs c- A^+^ than those in s-H vs c-H, and half of them, including *sptb* and *hbb*, were related to the blood circulation system. In addition, the cluster 2 and 4 contained *spen*, *gig2*, *huwe1*, *klf13* and *pikks*, which exhibited the most remarkable upregulation in clone F. The above results indicated that a persistent immune response might sustain in resistant clone H and F, while susceptible clone A^+^ had a long-term impact on the circulatory system.

### Distinct signaling pathways among the survivors of three gibel carp clones

In total, 1393, 2106 and 1617 DEUs were specifically up-regulated their expression levels in the survivors of clone A^+^, F and H, respectively in comparison with the control individuals (Fig. 4a; Additional file 6: Table S4). KEGG pathway analysis mapped these DEUs into 245, 276 and 252 pathways respectively (Additional file 7: Table S5). Among the top 30 enriched pathways, 26 pathways in resistant clone H were associated with infections, diseases, or immune-related pathways, such as “NF-kappa B signaling pathway”, “intestinal immune network for IgA production”, “Fc epsilon RI signaling pathway”, “RIG-I-like receptor signaling pathway”, and “toll-like receptor signaling pathway” (Fig. 3d). However, two-thirds of the top 30 pathways in s-F vs c-F belonged to “metabolism” or “biogenesis”, and only 7 pathways were involved in diseases or immune (Fig. 3e). In susceptible clone A^+^, 7 pathways involved in “cardiovascular diseases” or “circulatory system” were enriched among the top 30 pathways. In addition, 15 pathways were related to the infection, diseases, or immune (Fig. 3f).

Similarly, a total of 1049, 1451 and 1314 DEUs were identified as specifically down-regulated DEUs of clone A^+^, F and H, and mapped to 233, 249 and 243 pathways respectively (Additional file 6: Table S4 and Additional file 7: Table S5). Among the top 30 enriched pathways, we found 11 pathways related to “metabolism” in resistant clone H, 25 pathways involved in infections, diseases or immune in clone F, and 5 pathways associated with “circulatory system” or diseases in clone A^+^ (Fig. 3g-i). The differential top 30 pathways of specifically up-regulated or down-regulated DEUs in s-A^+^ vs c-A^+^, s-F vs c-F and s-H vs c-H revealed distinct immune responses activated or sustained in the survivors of three gibel carp clones.

### Differential expressions of IFN system genes and immunoglobulin genes among the survivors of three gibel carp clones

Among these DEUs specific to s-H vs c-H, 58 specifically up-regulated DEUs were annotated as IFN system genes, including host pattern recognition receptors (*lgp2*, *tlr4b*, *tlr22*, *nlrp3* and *nlrp12*), PRR-mediated IFN signal pathway (*stat1* and *stat2*), IFN regulatory factors (*irf7*), interferon-induced proteins or IFN antiviral effectors (*mx1*, *mx3*, *viperin*, *ifi27*, *ifi44*, *ifi56*, *gig1*, *gig2*, *gvinp1*, and *irgc*) (Table 3; Additional file 8: Table S6). We also found 43 specifically up-regulated DEUs belonged to immunoglobulin (Ig) superfamily, such as Ig light chain (*IgLλ* and *IgLκ*), Ig heavy chain (*IgM*, *IgD* and *IgZ*), and polymeric Ig receptor-like (*pIgRL*) (Table 3; Additional file 8: Table S6). The results suggest that both innate immune system and adaptive immune system persisted in activating in the survivors of resistant clone H after *Ca*HV challenge.

**Table 3:** Number of specifically up/down-regulated DEUs annotated as IFN system genes, immunoglobulins or myosins from s-H vs c-H, s-F vs c-F and s-A^+^ vs c-A^+^

Category		Gene Abbrev-iation	Number of up-regulated DEUs			Number of down-regulated DEUs		
			s-H vs c-H	s-F vs c-F	s-A^+^ vs c-A^+^	s-H vs c-H	s-F vs c-F	s-A^+^ vs c-A^+^
IFN system	host pattern recognition receptors	*lgp2*	3	1	0	0	0	1
		*tlr4b*	1	0	0	0	0	0
		*tlr22*	2	0	0	0	0	0
		*tlr7a*	0	0	0	0	1	0
		*nlrp3*	4	7	3	4	1	3
		*nlrp12*	4	4	1	0	0	0
		*ifnγr2*	0	3	0	0	0	0
	PRR-mediated IFN signal pathway	*stat1*	3	3	0	0	0	0
		*stat2*	3	0	0	0	0	0
	IFN regulatory factor	*irf3*	0	0	0	0	0	1
		*irf7*	4	0	0	0	0	1
	interferon- induced proteins or IFN antiviral 7effectors	*mx1*	4	6	4	3	3	3
		*mx3*	2	3	0	0	0	1
		*viperin*	4	0	0	0	0	2
		*ifi27*	2	0	0	0	0	0
		*ifi44*	4	6	0	0	0	1
		*ifi56*	1	0	0	0	0	0
		*gig1*	4	1	0	0	0	0
		*gig2*	3	0	0	0	0	0
		*gvinp1*	4	9	1	0	0	0
		*irgc*	2	0	0	0	0	0
		*isg15*	3	0	0	0	0	1
		*eif2ak2*	1	0	0	0	0	0
		*gbp*	0	2	0	0	0	0
		*traf2*	0	1	0	0	0	0
		*t2 bp*	0	1	0	0	0	0
		*ifitm1*	0	1	0	1	0	0
		*Ifitm3*	0	1	0	0	0	0
	interferon	*ifnγ1*	0	1	1	0	0	0
Total number			58	50	10	8	5	14
Immunoglobulin	immunoglobulin heavy chain	*IgM*	15	0	7	0	40	3
		*IgZ*	3	0	5	0	11	3
		*IgD*	1	0	1	0	0	0
	immunoglobulin light chain	*IgLλ*	13	0	19	4	66	4
		*IgLκ*	10	0	0	0	1	1
	immunoglobulin receptor-like	*pIgRL*	1	1	1	0	0	0
	immunoglobulin superfamily	*Igsf3*	0	0	0	1	0	2
Total number			43	1	33	5	118	13
myosin superfamily	myosin heavy chain	*myh7*	0	0	4	0	0	0
		*myh9*	0	0	1	0	2	1
		*myh10*	1	3	1	0	0	0
		*myh11*	0	0	0	0	1	0
		*myha*	0	0	2	0	0	0
		*myhb*	0	0	1	0	2	0
		*myo3b*	0	0	1	0	0	0
		*myo5a*	0	0	0	1	0	0
		*myo9b*	0	1	0	0	1	0
		*myo10*	0	1	0	0	1	0
		*myo18a*	0	1	1	0	0	0
		*myo18b*	0	0	0	1	0	0
	myosin light chain	*myl1*	0	0	0	0	1	0
		*myl2*	0	0	2	0	2	0
		*myl3*	0	0	3	0	1	0
		*myl10*	0	0	1	0	1	0
		*mylk*	2	0	0	1	0	0
Total number			3	6	17	3	12	1

Similarly, 50 specifically up-regulated DEUs identified from s-F vs c-F were classified into IFN system genes, such as *ifnγ1*, *ifnγr2*, *ifitm*, *gbp*, *mx1*, *mx3* and so on (Table 3; Additional file 8: Table S6). Interestingly, the dynamic expression patterns of Ig genes in moderate resistant clone F were completely different from the high resistant clone H. A total of 118 specifically down-regulated DEUs were annotated as members of Ig superfamily, including *IgLλ* (66), *IgLκ* (1), *IgM* (40), and *IgZ* (11). The above results indicate that IFN immune system still retained high expression levels, while the adaptive immune system might be impaired in the survivors of clone F.

In comparison with control individuals, the expression changes of IFN system genes and Ig genes in the survivors of susceptible clone A^+^ exhibited distinct pattern from those in the resistant clone H and F. Ten specifically up-regulated and 14 specifically down-regulated DEUs were annotated as IFN system genes, including *lgp2*, *nlrc3*, *irf3*, *irf7*, *isg15*, *mx1*, *mx3*, or *viperin* (Table 3; Additional file 8: Table S6). We also identified 33 and 13 DEUs annotated as members of Ig superfamily which were specifically up-regulated or down-regulated their expression levels from s-A^+^ vs c- A^+^. Moreover, a total of 17 specifically up-regulated DEUs belonged to myosin superfamily (*myl2*, *myl3*, *myl10*, *myha*, *myhb*, *myh7*, *myh9*, *myh10*, *myo3b*, and *myo18a*), which all mapped into the pathways of “circulatory system” or diseases in circulatory system (Fig. 3f).

**Fig. 5:**
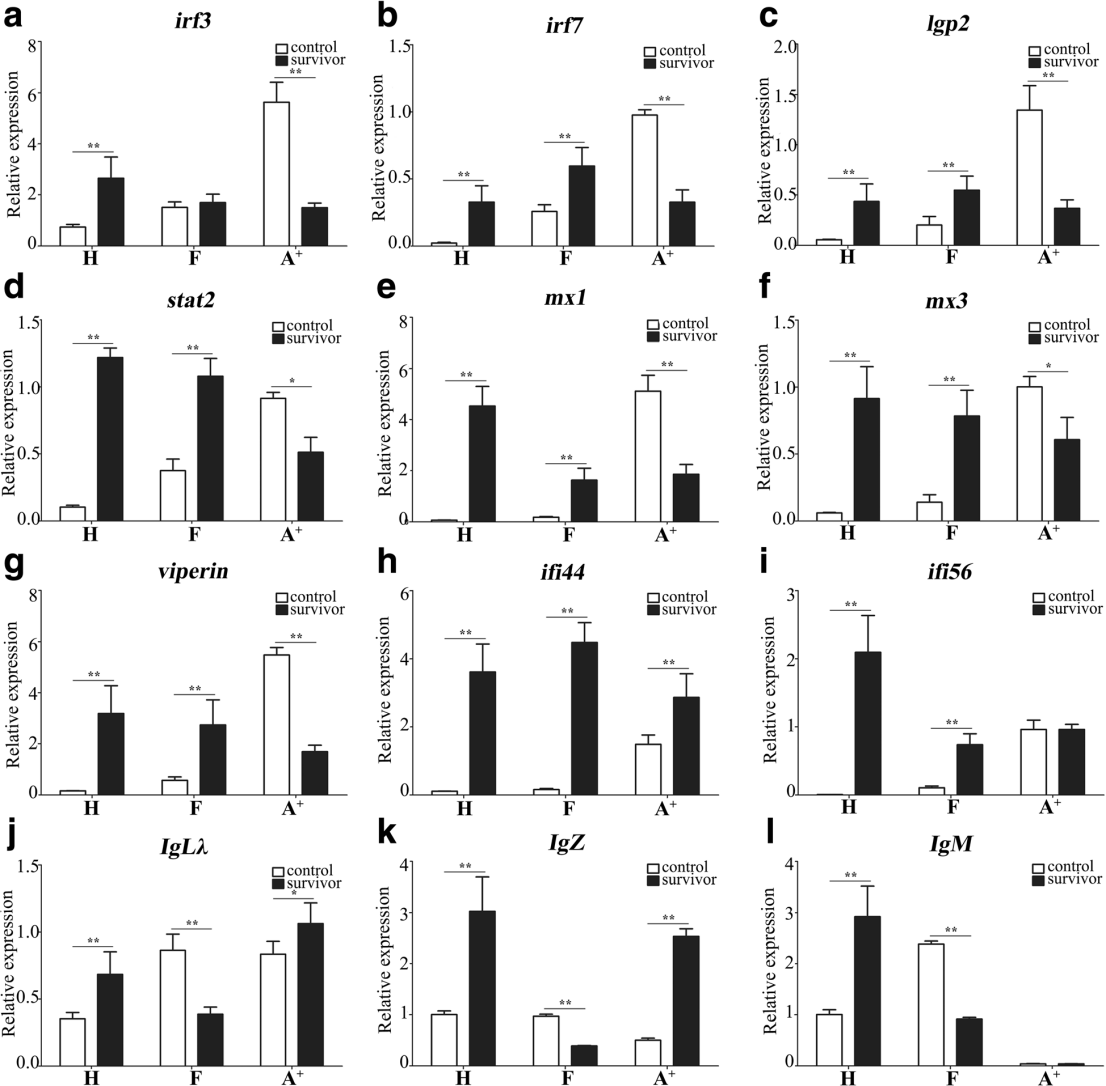
Verification of DEUs by qPCR. The qPCR analyses of nine IFN system genes (*irf3*, *irf7*, *lgp2*, *stat2*, *mx1*, *mx3*, *viperin*, *ifi44*, and *ifi56*) (a-i) and three immunoglobulins (*IgLλ*, *IgZ* and *IgM*) (j-l) in head kidney of gibel carp clone A^+^, F and H. *eef1a1l1* was used as the normalizer. Gene expression levels are relative to those of clone H control individuals. Each bar represents mean ± standard deviation (SD) (n = 3). Asterisks indicate the significant differences between control and survivor (*: *p* < 0.05 and **: *p* < 0.05)

To validate the differential expressions among the survivors of three gibel carp clones, 12 genes, including nine IFN system genes and three immunoglobulins, were selected for qPCR analysis (Fig. 5). Except *ifi44* and *ifi56*, the other 7 IFN system genes were all down-regulated their expressions in the survivors of susceptible clone A^+^ in comparison with control individuals, while the 9 genes were all up-regulated in the survivors of the resistant clone H and F. Consistent with the transcriptome results, immunoglobulins (*IgLλ*, *IgZ* and *IgM*) were down-regulated in s-F vs c-F, while up-regulated in s-H vs c-H. In susceptible clone A^+^, the expression of *IgM* was very low. Compared to control, *IgM* and *IgLλ* had little change of their expression, while *IgZ* were up-regulated in the survivors of clone A^+^. Significantly, most of these genes displayed relatively higher up-regulated folds in clone H than those in clone F. qPCR analysis confirmed the differential expressions of IFN system genes and immunoglobulins among the survivors of three gibel carp clones.

### Myosin and immunoglobulin identified as positively or negatively correlated genes to *Ca*HV viral loads respectively

**Fig. 6:**
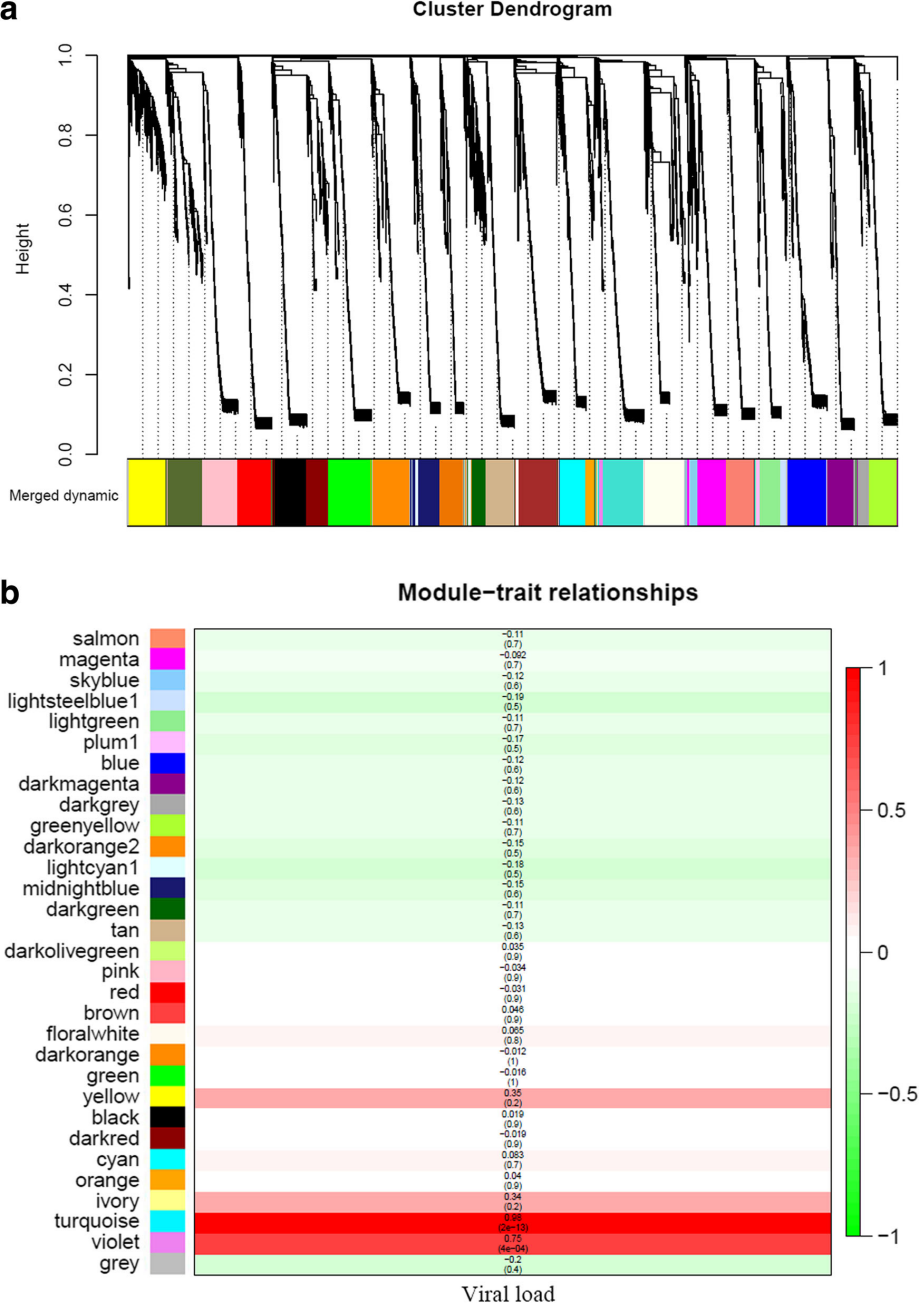
WGCNA identification of gene modules correlated with resistance or susceptibility traits of gibel carp. **a** The clustering dendrogram of DEUs. Branches represent the co-expressed genes in modules, shown in the colorbar below the dendrogram. **b** Heatmap of correlations between modules and viral loads. Each cell contains the corresponding correlation and *p*-value. Positive or negative correlations are denoted in red or green respectively

Weighted gene correlation network analysis (WGCNA) was employed to further investigate the relationship between the expression changes of DEUs (Additional file 2: Table S2) and viral loads in the survivors of three gibel carp clones (Fig. 1). Based on the correlation coefficients of DEUs, 31 distinct co-expression modules were identified and labeled by randomly assigned colors (Fig. 6a). These modules were then correlated with resistance or susceptibility traits by calculating the correlations (Fig. 6b). The turquoise and lightsteelblue1 were the most significantly positively or negatively correlated with the viral loads in the survivors respectively (Fig. 6b; Additional file 9: Table S7). The turquoise module contained 146 DEUs, 12 of which were annotated as members of myosin superfamily (*mylh2*, *mylh3*, *mylh10*, *myha*, *myh7*, *myo3b*, and *myo18a*) (Additional file 9: Table S7). The lightsteelblue1 modules included only 35 DEUs, 11 of which belonged to Ig superfamily (*IgLλ*, *IgLκ* and *IgM*) (Additional file 9: Table S7). The above results showed that myosin and immunoglobulin were positively or negatively correlated to *Ca*HV viral loads respectively.

## Discussion

Owing to its highly intensive farming, the culture industry of crucian carp has suffered enormous economic losses caused by *Ca*HV. In previous study, we screened the resistant clone H as the core breeding population and revealed the underlying antiviral mechanisms in gibel carp, activating IFN system and suppressing complements [29]. In this study, we confirmed that the resistant clone H possessed not only stronger resistance but also higher tolerance to *Ca*HV (Fig. 1b). The GO and KEGG analysis showed that the genes related to circulatory and immune system were activated in the survivors of all three gibel carp clones after *Ca*HV challenge (Figs. 2 and 3 and Tables 1 and 2). Significantly, we revealed the distinct signaling pathways (Fig. 3) and differential gene changes of innate immune gene (IFN system genes) and adaptive immune system (Ig genes) among three gynogentic gibel carp clones (Fig. 5 and Table 3). Moreover, we identified myosins and Igs as positively or negatively correlated genes to *Ca*HV viral loads by WGCNA respectively (Fig. 6).

Virus invasion induces a chain of immune responses in host. In fish, IFN response plays a vital role in the first line of host defense against virus infection [34, 36, 37, 41–47]. After *Ca*HV infection, the up-regulation folds of eight IFN system genes in three gibel carp gynogenetic clones, including *retinoic acid-inducible gene I* (*RIG-I*), *laboratory of genetics and physiology 2* (*LGP2*), *IFN regulatory factors* (*IRFs*) (*IRF1*, *IRF3*, *IRF7*, and *IRF9*), *Mx*, and *viperin*, were related to their resistance ability to *Ca*HV, progressively increasing from susceptible clone A^+^, moderate resistant clone F to resistant clone H at 1 dpi [40]. However, immune inhibitory receptor *DICPs* (*diverse Ig domain-containing proteins*) showed the opposed expression patterns [48]. Thus, gibel carp might recognize *Ca*HV products by RIG-I and LGP2, then activate mitochondrial antiviral signaling protein (MAVS)-dependent pathway and induce the expression of IRFs (*IRF1*, *IRF3*, *IRF7*, and *IRF9*), IFNs and interferon stimulated gene (*Mx* and *viperin*) after *Ca*HV infection [40]. Similarly, a lot of IFN system genes were up-regulated their expression in the survivors of resistant clone H and F (Table 3), and most of them displayed relatively higher up-regulated folds in clone H than those in clone F (Fig. 5). Taken together our previous results [29, 40, 48], we believe that stronger IFN response were triggered and persisted in gibel carp resistant clone H after *Ca*HV challenge. The above results also confirmed the critical function of IFN system against viral infection in teleost [36, 42, 45, 49, 50]. Conversely, the dynamic expression changes in susceptible clone A^+^ are completely different. Many IFN system genes, such as *irf3*, *irf7*, *lgp2*, *stat2*, *mx1*, *mx3*, *viperin*, and so on, were down-regulated their expressions in the survivors of susceptible clone A^+^ in comparison with control individuals (Table 3 and Fig. 5). Various viruses have been shown to inhibit the IFN signaling through different escape strategies to promote virus replication and release [51–54]. On the other hand, host might adopt a negative feedback mechanism to respond excessive and long-term IFN stimulation, which prevents inappropriate antiviral reactions and autoimmunity [55–57]. For example, compared to wildtype (WT), the expression levels of IFN-signal modulators (*stat1b*, *stat2*, *mad5*, *mita*, *irf3*, and *ifr9*), ISGs (*mx*, *gig1a*, *isg15*, and *isg56*) and *ifnλ* were down-regulated in IFNd-Tg fish, a transgenic medaka (*Oryzias latipes*) that constitutively overexpressed the *ifnd* gene [57]. Different from the immediately up-regulated expression of IFN system genes in resistant clone H at 1 dpi, most of them were up-regulated at 3–5 dpi in susceptible clone A^+^ [40], indicating that the delay of IFN response might affect the expression of IFN system genes in the survivors of susceptible clone A^+^. In addition, among the top 30 enriched pathways of down-regulated DEUs, 11 pathways in resistant clone H belonged to “metabolism”, but only 1 and 2 pathways involved in “metabolism” were found in clone A^+^ and F (Fig. 3g-i). The result indicates that the up-regulation of IFN may down-regulated metabolism, consistent with the phenomenon in mouse [58, 59].

Interestingly, myosins were identified as positively co-expression module correlated to *Ca*HV viral loads by WGCNA analysis (Fig. 6 and Additional file 9: Table S7). Myosins are composed of a large superfamily of proteins with similar structures and act as actin-based motor [60]. Viral infections induces dramatic cytoskeletal reorganization, and virion movement is mediated by myosin motors [61–64]. For example, Myh9 can transport infectious viral RNA from cell to cell to accelerate intercellular viral spread [65–67], and can inhibit RhoA signaling, resulting in increased virus release and more viral spread [68]. Moreover, the microRNAs (miRNAs) encoded by the introns of some myosin genes can suppress the expression of *IFNλ2* and *IFNλ3* and type I IFN receptor chain *IFNAR1* to support viral persistence [69, 70]. In this study, we observed many myosin genes, including *myh9*, were up-regulated in the survivors of susceptible clone A^+^ (Table 3; Additional file 8: Table S6), consistent with the down-regulated expression of IFN system genes (Table 3; Additional file 8: Table S6). Additionally, few myosin genes were identified as up-regulated DEUs in the survivors of resistant clone H and F in which the expression of IFN system genes were up-regulated (Fig. 5; Table 3; Additional file 8: Table S6). The above results reveal the intrinsic connections among myosins, IFN response and *Ca*HV viral loads which awaits further investigation.

Besides the distinct expression changes of IFN system genes, we also observed the differential reaction of adaptive immunity to *Ca*HV among three gynogentic clones (Fig. 5; Table 3; Additional file 8: Table S6). Furthermore, we identified Igs as negatively co-expression module correlated to *Ca*HV viral loads (Fig. 6; Additional file 8: Table S7). Like mammals, teleost adaptive immune systems include recombination activating gene (RAG), Igs, T cell receptors (TCR) and major histocompatibility complex (MHC) molecules [39, 71]. Igs are specifically produced by B lymphocytes and play pivotal roles in adaptive immunity. They have evolved highly diversified molecules toward recognizing a remarkably large number of antigens to mediate the intracellular neutralization of viruses [72–74]. In teleost, several Igs, such as IgL, IgM, IgD and IgZ, were identified [39, 75–78]. In this study, 43 and 33 up-regulated DEUs annotated as members of Ig superfamily were screened from resistant clone H and susceptible clone A^+^ respectively, while a total of 118 down-regulated DEUs were classified into Ig superfamily in clone F (Table 3; Additional file 8: Table S6). The host relies on the synergistic action of innate immune system and adaptive immune system, and simultaneously activates the resistance mechanism and disease tolerance strategy to reduce the amount of viruses in the host and the damage of the host parenchyma [79]. Disease tolerance does not exert a direct negative effect on the host viral loads and depends on tissue damage control mechanisms which also regulated by innate and adaptive components of the immune system [80, 81]. The activation of IFN signaling can not only inhibit viral replication and release [82–84], but also activate lymphocytes, natural killer (NK) cells, and macrophages [85]. IFN is believed acting as the bridge between innate and adaptive immunity [86, 87]. The activation of both innate immune system and adaptive immune system might be the reason for the highest resistant ability of clone H. Interestingly, the survivors of moderately resistant clone F kept the activation of innate immune system but the impaired adaptive immune system (Table 3; Additional file 8: Table S6). This similar pattern of immune response was also been found in gilthead seabream (*Sparus aurata* L.) infected with lymphocystis disease virus (LCDV) [88]. Among the top 30 pathways enriched by DEUs specifically up-regulated their expression levels in the survivors of clone F, two-thirds of pathways belonged to “metabolism” or “biogenesis” (Fig. 3e) which suggested that clone F might actively repair the impairment, including adaptive immune system. Along with the divergence of cyclostomes and cartilaginous fish, adaptive immunity emerged in vertebrate around 450 million years ago [89, 90]. However, it is believed that the innate immunity plays the dominant role in resistance to viral infection in teleost, due to the highly specialized immune responses (which means limited recognition of pathogens), low antibody affinity, slow antibody response, and weak memory response in fish adaptive immunity [39, 71, 91]. Therefore, the clone F showed moderate resistant ability to *Ca*HV, which is weaker than that of clone H (IFN system genes and Ig genes both up-regulated their expression) and higher than that of clone A^+^ (IFN system genes down-regulated and Ig genes up-regulated their expression). Moreover, susceptible clone A^+^ failed to trigger IFN antiviral response but activated myosins to increase the virus release and spread (Table 3; Additional file 8: Table S6), which resulted in the loss of battle between host and *Ca*HV and severely impaired circulatory system (Fig. 5f). Thus, the further analysis are necessary to confirm the differential innate and adaptive immune responses, to reveal the association among IFN system, myosin, Igs and disease resistance/susceptibility among three gibel carp clones.

## Conclusions

The current study represents a paradigm of differential innate and adaptive immune reactions among fishes with distinct resistance to virus. Our results suggest that the differential regulation mechanism between innate and adaptive immunity may be the key reason for the distinct resistance to *Ca*HV among the three gibel carp gynogenetic clones. The establishments of three gibel carp gynogenetic clones with distinct resistances to *Ca*HV will provide a model to definitely clarify the regulatory interactions among IFN signaling, Igs, myosins and virus in teleost. In addition, our studies will be helpful to the disease-resistance breeding of gibel carp.

## Methods

### *Ca*HV infection and sample collection

Six month old gibel carps were collected from the GuanQiao Experimental Station, Institute of Hydrobiology, Chinese academy of sciences. The *Ca*HV challenge experiments and the confirmation of control individuals with *Ca*HV free were performed as previously described [29, 40]. The mortalities caused by *Ca*HV subsided after 14 dpi, and the *Ca*HV challenge experiment was terminated at 28 dpi. Head-kidney tissues were collected from control fishes and healthy survivors at 28 dpi. All samples were preserved in RNAlater (QIAGEN) and stored at − 20 °C for nucleic acid extraction. All procedures in this study were performed following the ethical requirements of Animal Care and Use Committee of Institute of Hydrobiology, Chinese Academy of Sciences to minimize animal suffering. After deep and overdosed anesthesia with styrylpyridine (30-50 mg/L; aladdin, China), the fish were euthanized by immediately cutting off the spinal cord adjacent to the head.

### *Ca*HV detection and quantification

Three control individuals, diseased individuals or survivors of each clone were selected to evaluate viral load. Total DNA was isolated using DNA extraction kit (Promega, USA) following the manufacturer’s protocol. Five genes, including *hel*, *cap*, *ung*, *dut*, and *tmk*, were selected to detect the existence of *Ca*HV. The primers were designed according to the *Ca*HV genome sequence (KU199244) (Additional file 10: Table S8). Amplification was performed in 25 μL PCR mixture containing 100 ng of template DNA, 0.2 mM of each primer, 1.5 mM of MgCl_2_, 0.2 mM of each dNTP, 2.5 μL of 10× *Taq* buffer, and 1 unit of *Taq* DNA polymerase (Thermo Fisher Scientific, USA). The PCR amplification was performed with an initial denaturation for 3 min at 95 °C, followed by 40 cycles of denaturation for 30 s at 95 °C, annealing for 30 s at 60 °C, extension for 45 s at 72 °C and a final extension for 5 min at 72 °C. The PCR products were analyzed by agarose gel electrophoresis. β-actin was selected as reference gene (Additional file 10: Table S8). Viral load were evaluated by real-time PCR as previously described [29, 40, 92]. Briefly, a 637 bp fragment of *Ca*HV helicase gene was amplified, purified and inserted into the pMD18-T plasmid to produce pMD-*Ca*HV (3329 bp). Then, pMD-*Ca*HV was diluted into 10-fold dilution series and used as the standard template of *Ca*HV to preform quantitative real-time PCR as previously described [92].

### RNA sequencing, assembly, annotation and differential expression analysis

To avoid the individual differences in virus susceptibility, head-kidney tissues from three survivor with similar average viral load (10 particles/ng DNA) of each clone were collected for RNA-Seq. The RNA isolation, library construction and sequencing were performed as previously described [29]. Briefly, the total RNAs were isolated by using SV Total RNA isolation System (Promega, USA) and assessed their quantity and quality by Agilent 2100 Bioanalyzer. The library construction, Hiseq 4000™ sequencing, De novo assembly, screening and functional annotation of DEUs were accomplished by Beijing Genomics Institute (BGI), China. Firstly, total RNAs were digested by Dnase I (NEB), purified by oligo-dT beads (Invitrogen) and fragmented with Fragment buffer (Ambion). Then, the first and second strand cDNAs were synthesized successively by First Strand Master Mix and Second Strand Master Mix (Invitrogen) respectively, and purified and combined with End Repair Mix. After purified and mixed with A-Tailing Mix, Adenylate 3′Ends DNA, and Adapter and Ligation Mix, DNA fragments between 300 bp–350 bp were selected and enriched. The final libraries were quantified by real-time qPCR (TaqMan Probe) and were amplified on cBot to generate the cluster on the flowcell. Finally, the amplified flowcells were sequenced on the Illumina Hiseq platform to generate 150 bp single-end reads. The raw sequencing reads were filtered to remove the reads with low quality or adaptors to obtain clean reads, which then were assembled into unigenes by Trinity [93] and Tgicl [94]. Subsequently, these unigenes were annotated with National Center for Biotechnology Information (NCBI) non-redundant protein (NR), NCBI non-redundant nucleotide (NT), GO, Clusters of Orthologous Groups of proteins (COG), KEGG, InterPro and Swiss-Prot database using Blast2GO [95], and InterPro database using InterProScan5 [96].

Clean reads were mapped to unigenes using Bowtie2 [97] and the expression levels of unigenes were calculated by RSEM (RNA-Seq by Expectation Maximization) [98] and DEUs were identified with NOIseq based on noisy distribution model [99]. The threshold to judge the significant difference was defined as “relative change ≥ 2 and probability ≥ 0.8”. KEGG enrichment analysis of DEUs was performed by phyper, a function of R. False discovery rate (FDR) was used to determine the threshold of *p* value and KEGG terms (FDR ≤ 0.01) were considered significantly enriched. Venn and heatmap diagram were generated by using jvenn [100] and MeV, respectively.

### Weighted gene correlation network analysis (WGCNA)

To figure out the relationship between DEUs and viral loads, WGCNA was carried out by the WGCNA R package [101, 102]. Expression correlation coefficients of all DEUs were firstly calculated and a suitable soft threshold was chosen to build gene networks by using the scale-free topology criterion. Subsequently, based on the hierarchical clustering built by the topological overlap matrix and using the dynamic tree cut method, the co-expression modules were identified (minModuleSize = 30 and mergeCutHeight = 0.25). Then, the module eigengenes (MEs) were determined by using the expression data within each module and the module-trait relationships were calculated based on the correlations between MEs and viral loads of samples.

### Quantitative reverse transcription PCR (qPCR)

The head kidney tissues of three control individuals or survivors of each clone were selected to perform qPCR. The total RNA isolation and first-strand cDNAs synthesis were performed according to the previous description [29, 40, 103]. According to the sequences of the de novo assembly data (SRP096800), the primers were designed (Additional file 10: Table S8). qPCR was performed in the CFX96™ Real-Time PCR System (Bio-Rad) by using iTaq™ Universal SYBR® Green Supermix (Bio-Rad, USA). The reaction system, PCR protocols and negative control selection were also performed as described previously [29, 40]. All samples were analyzed in triplicate, and the relative expression levels were calculated by using the 2^-ΔΔCT^ method. Tukey’s test was calculated with SPSS software (SPSS Inc.) for statistical analysis. A probability (p) of < 0.05 was considered statistically significant. According to the screening results of optimal reference gene [40], *eukaryotic translation elongation factor 1 alpha 1, like 1* (*eef1a1l1*) (M value = 0.74 < 1.5), was used as the normalizer for qPCR.

## Additional files

Additional file 1: **Table S1.** Summary statistics of sequencing data. Quality of sequencing reads, transcripts and unigenes are shown respectively. The number 1, 2 and 3 represent three replicate samples of each group. (XLSX 12 kb)

Additional file 2: **Table S2.** Differentially expressed unigenes (DEUs) (Probability ≥0.8 and relative change ≥2) in s-A^+^ vs c-A^+^, s-F vs c-F and s-H vs c-H. Lists of DEUs include Unigene ID, Length, FPKM, log_2_ fold change, *P* value, up- or down-regulation and annotation. (XLSX 1172 kb)

Additional file 3: **Figure S1.** The numbers of differentially expressed unigenes (DEUs) from s-A^+^ vs c-A^+^, s-F vs c-F and s-H vs c-H. The red and green bars indicate up- and down-regulated DEUs respectively. (TIF 89 kb)

Additional file 4: **Figure S2.** KEGG pathway enrichment analysis. a-c Top 10 enriched KEGG pathways of DEUs from s-H vs c-H (a), s-F vs c-F (b) and s-A^+^ vs c-A^+^ (c). The x-axis indicates the rich factor of each pathway and y-axis shows pathway. The color and size of dot indicates Q value and the number of DEUs assigned to the corresponding pathway respectively. (TIF 1300 kb)

Additional file 5: **Table S3.** Enriched KEGG pathways in s-A^+^ vs c-A^+^, s-F vs c-F and s-H vs c-H. KEGG pathway description, number of DEUs assigned to the corresponding pathway, P value, Q value, Pathway ID, KEGG function classification, annotated KO ID of DEUs and unigene ID assigned to the corresponding pathway are shown. (XLSX 153 kb)

Additional file 6: **Table S4.** The common and unique differentially expressed unigenes (DEUs) in s-A^+^ vs c-A^+^, s-F vs c-F and s-H vs c-H. List of DEUs includes Unigene ID, FPKM, log_2_ fold change, up- or down-regulation and annotation. (XLSX 810 kb)

Additional file 7: **Table S5.** Enriched KEGG pathways of DEUs commonly up- and down-regulated in three clones and specifically up- and down-regulated in s-A^+^ vs c-A^+^, s-F vs c-F and s-H vs c-H. KEGG pathway description, number of DEUs assigned to the corresponding pathway, P value, Q value, Pathway ID, KEGG function classification, annotated KO ID of DEUs and unigene ID assigned to the corresponding pathway are shown. (XLSX 188 kb)

Additional file 8: **Table S6.** Up−/down-regulated DEUs annotated as IFN system genes, immunoglobulins and members of myosin superfamily in s-A^+^ vs c-A^+^, s-F vs c-F and s-H vs c-H. List of DEUs includes Unigene ID, FPKM, log_2_ fold change, up- or down-regulation and annotation. (XLSX 67 kb)

Additional file 9: **Table S7.** DEUs in the turquoise and lightsteelbiue1 modules. List of DEUs includes Unigene ID, Module, Correlation, P value and annotation. (XLSX 62 kb)

Additional file 10: **Table S8.** Primers used in this study. (XLSX 11 kb)

## Abbreviations

*alas2*: Delta-aminolevulinate synthase
*alox15*: Arachidonate 15-lipoxygenase
*ank1*: Ankyrin 1
*birc6*: Baculoviral IAP repeat-containing protein 6
*blvrb*: Flavin reductase
*Ca*HV: *Carassius auratus* herpesvirus
*cahz*: Carbonic anhydrase
*cap*: Capsid triplex subunit 2
COG: Clusters of Orthologous Groups of proteins
*cpox*: Coproporphyrinogen oxidase
DEUs: Differentially expressed unigenes
*DICP*: Diverse Ig domain-containing protein
dpi: Days post injection
*dut*: Deoxyuridine triphosphatase
*eef1a1l1*: Eukaryotic translation elongation factor 1 alpha 1, like 1
*fam210b*: Family with sequence similarity 210 member B
*fech*: Ferrochelatase
*gbp*: Guanylate-binding protein
*gig*: Grass carp hemorrhagic virus-induced gene
GO: Gene Ontology
*gvinp1*: Interferon-induced very large GTPase 1
*hba*: Hemoglobin alpha
*hbb*: Hemoglobin beta
*hel*: Helicase-primase helicase subunit
*hemge*: Hemogen
*hmbs*: Hydroxymethylbilane synthase b
*huwe1*: E3 ubiquitin-protein ligase huwe1
*ifi*: Interferon-induced protein
*ifitm1*: Interferon-induced transmembrane protein 1
IFN: Interferon
*ifnγ*: Interferon gamma
*IgD*: Immunoglobulin D
*IgLκ*: Immunoglobulin kappa
*IgLλ*: Immunoglobulin lambda
*IgM*: Immunoglobulin M
Igs: Immunoglobulins
*IgZ*: Immunoglobulin Z
*irf*: Interferon regulatory factor
IRFs: IFN regulatory factors
*irgc*: Interferon-inducible GTPase 5
*isg15*: Interferon-stimulated gene 15
ISGs: Interferon stimulated genes
KEGG: Kyoto Encyclopedia of Genes and Genomes
*klf*: Kruppel-like factor
LCDV: Lymphocystis disease virus
*lgp2*: Laboratory of genetics and physiology 2
MAVS: Mitochondrial antiviral signaling protein
*mfrn1*: Mitoferrin-1
MHC: Major histocompatibility complex
miRNAs: microRNAs
*mt1*: Metallothionein
*mx*: Interferon-induced GTP-binding protein
*mycbp2*: E3 ubiquitin-protein ligase mycbp2
*myh7*: Myosin-7
*myha*: Myosin heavy chain a
*myhb*: Myosin heavy chain b
*myl*: Myosin light chain
*myo18a*: Myosin-XVIIIa
*myo3b*: Myosin-IIIb
NCBI: National Center for Biotechnology Information
*nf1*: Nuclear factor I
NK: Natural killer
*nlrp*: NACHT, LRR and PYD domains-containing protein
NR: NCBI non-redundant protein
NT: NCBI non-redundant nucleotide
*pIgRL*: Polymeric immunoglobulin receptor-like
*pikks*: Phosphatidylinositol 3-kinase-related kinase
*pla2g4b*: Cytosolic phospholipase A2 beta
RAG: Recombination activating gene
RIG-I: Retinoic acid-inducible gene I
RSEM: RNA-Seq by Expectation Maximization
*slc10a4*: Sodium/bile acid cotransporter 4
*slc4a1*: Band 3 anion transport protein
*spen*: Msx2-interacting protein
*sptb*: Spectrin beta
*stab1*: Stabilin-1
*stat*: Signal transducer and activator of transcription
TCR: T cell receptors
*tfr*: Transferrin receptor
*tlr*: Toll-like receptor
*tmk*: Thymidylate kinase
*ung*: Uracil-DNA glycosylase
*urod*: Uroporphyrinogen decarboxylase
*vps13c*: Vacuolar protein sorting-associated protein 13C
*wdfy4*: WD repeat and FYVE domain-containing protein 4
WGCNA: Weighted gene correlation network analysis
WT: Wildtype
