# Supplementary figures and images for: Differential expression of innate and adaptive immune genes in the survivors of three gibel carp gynogenetic clones after herpesvirus challenge
Source: BMC Genomics. 2019 May 28;20:432. doi: 10.1186/s12864-019-5777-z (PMC6540555; doi:10.1186/s12864-019-5777-z)

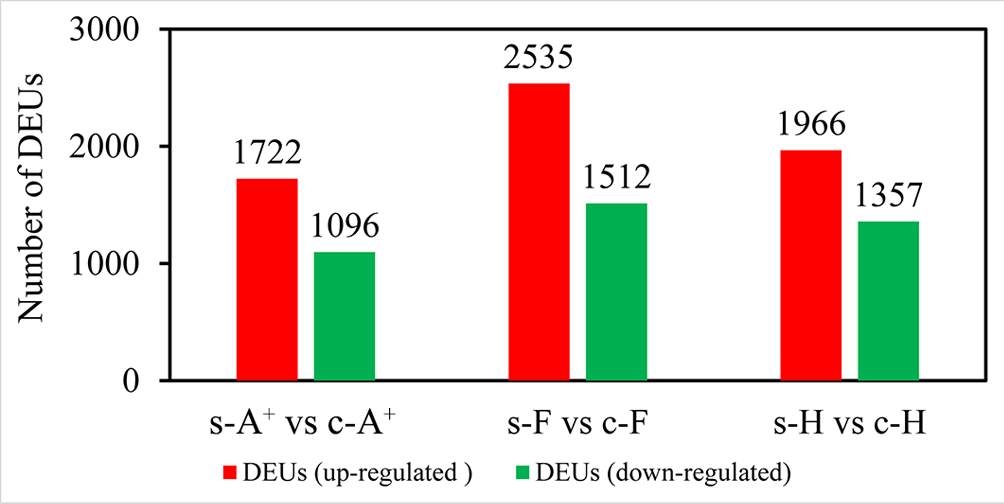

Supplement: Supplementary file 3 — Figure S1. The numbers of differentially expressed unigenes (DEUs) from s-A+ vs c-A+, s-F vs c-F and s-H vs c-H. The red and green bars indicate up- and down-regulated DEUs respectively. (TIF 89 kb) [file 12864_2019_5777_MOESM3_ESM.tif]

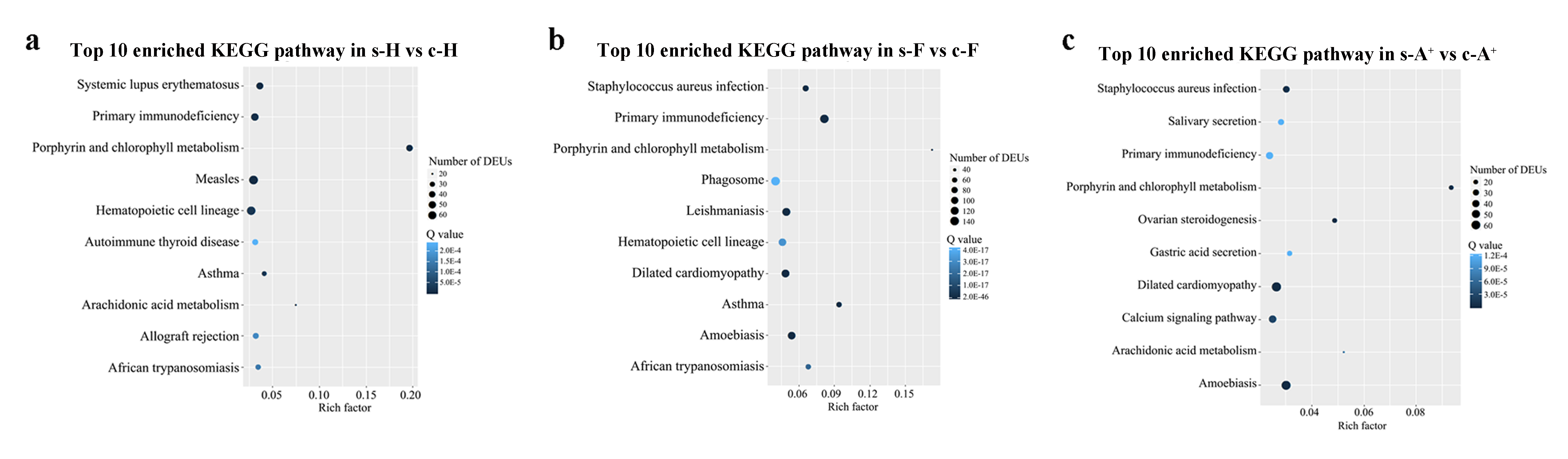

Supplement: Supplementary file 4 — Figure S2. KEGG pathway enrichment analysis. a-c Top 10 enriched KEGG pathways of DEUs from s-H vs c-H (a), s-F vs c-F (b) and s-A+ vs c-A+ (c). The x-axis indicates the rich factor of each pathway and y-axis shows pathway. The color and size of dot indicates Q value and the number of DEUs assigned to the corresponding pathway respectively. (TIF 1300 kb) [file 12864_2019_5777_MOESM4_ESM.tif]
